# Age and Gender Adjusted Comparison of Clinical Features between Severe Cases Infected with H7N9 and H1N1pdm Influenza A in Jiangsu Province, China

**DOI:** 10.1371/journal.pone.0120999

**Published:** 2015-03-27

**Authors:** Xiang Huo, Ke Xu, Qigang Dai, Xian Qi, Huiyan Yu, Changjun Bao

**Affiliations:** Department of Acute Infectious Disease, Jiangsu Provincial Center for Disease Control and Prevention, Nanjing, China, 210009; The University of Tokyo, JAPAN

## Abstract

**Background:**

Influenza H7N9 and H1N1pdm can cause severe human infections. It is important to investigate the distinguishing clinical features between these two diseases. Several studies have compared the differences in general, however, age and gender adjusted comparisons may be more useful and informative to the health professionals.

**Methods:**

A total of 184 severe H1N1pdm patients and 37 severe H7N9 patients from Jiangsu Province were included in this analysis to perform age and gender adjusted comparison of clinical features.

**Results:**

After adjusting age and gender, no significant differences in chronic medical conditions or treatment were found between severely ill patients with H7N9 and H1N1pdm. Severely ill patients with H7N9 had significantly longer interval from onset of illness to neuraminidase inhibitor treatment and to death. They were more likely to have complications such as acute respiratory distress syndrome (ARDS), liver and renal dysfunctions, and had a significantly higher risk of death.

**Conclusion:**

Our results suggests that age and gender should be adjusted as important confounding factors when comparing the clinical features between severe H7N9 and H1N1pdm patients to avoid any misunderstanding regarding the differences between these two diseases particularly in terms of clinical severity and prognosis.

## Introduction

Novel avian-origin influenza A(H7N9) virus was identified in March 2013 in China, and continue to cause human infections [1]. As of March 18, 2014, a total of 385 laboratory-confirmed human cases with avian influenza A(H7N9) virus infection were reported in Mainland China, and around 12% of them were from Jiangsu Province located in the eastern China (from China Information System for Disease Control and Prevention, http://10.249.1.170/). The first case as well as most of the cases reported in 2013 came from Yangtze River Delta Region, which consists of Jiangsu Province, Zhejiang Province and Shanghai Municipality[2]. Most of the H7N9 patients were severely ill; of them, 76.6% were admitted to an intensive care unit (ICU) [3]. To date, there is no evidence of sustained human-to-human transmission. In contrast, the clinical severity of pandemic A(H1N1) 2009 (H1N1pdm), a swine-origin reassortant virus which is able to transmit efficiently among individuals, were much milder with a case-ICU rate of 7.9–75 cases per 100,000 infections [4]. Even though, a large quantity of severe H1N1pdm patients was reported due to the widespread transmission of virus in human population. Owing to improve our understanding regarding the distinguishing clinical features of severe patients caused by these two viruses, we aimed to compare the key variables of laboratory-confirmed human cases of influenza H7N9 and H1N1pdm in Jiangsu Province of China.

## Materials and Methods

### Participants

All laboratory-confirmed cases infected with H7N9 and H1N1pdm are reported through a national system for reporting of notifiable infectious diseases [5]. Demographic, epidemiological and basic clinical data for severe patients infected with H7N9 and H1N1pdm were collected on standardized forms by local CDC staff or trained clinical doctors in Jiangsu Province and reported through this system. Information used in the present analysis included the age, sex, weight, height, underlying medical disorders associated with an increased risk of influenza complications [6]; dates of illness onset, medical consultation, hospital and ICU admission, death; treatment and clinical outcome. In total, 184 severe H1N1pdm patients from Jiangsu Province (illness onset during the 2009 H1N1 pandemic, i.e. from June of 2009 to February 2010) and 37 severe H7N9 patients (as of 8 March 2014) with detailed information were included in this analysis.

### Ethic statement

The National Health and Family Planning Commission decided that the collection of data from cases of both H7N9 and H1N1pdm was part of the public health investigation of emerging outbreak, and thus the investigation was exempt from institutional review board assessment [5]. Demographic, epidemiological and basic clinical data for severe patients infected with H7N9 and H1N1pdm were collected on standardized forms by local CDC staff or trained clinical doctors in Jiangsu Province and then reported to Jiangsu Provincial CDC and China CDC through a national system for reporting of notifiable infectious diseases. Jiangsu Provincial CDC is responsible for the checking and monitoring of the reporting information and will investigate directly if necessary. All the authors came from Jiangsu Provincial CDC and were in charge of the influenza surveillance and emergency response. The data set was not anonymized in the reporting system but was anonymized before data analysis.

### Definitions of severe cases

According to the Diagnosis and Treatment Scheme published by the National Health and Family planning commission of China, H1N1pdm patients who met either of the following criteria were defined as severe infections: 1) respiratory failure, 2) septic shock, 3) multiple organ dysfunction or 4) any other severe clinical condition required close monitoring in intensive care unit (ICU); and in the context of H7N9, patients with pneumonia and either respiratory failure or any other organ dysfunction were considered as severe infections. The criteria of severe infections with these two different influenza strains are comparable. Furthermore, the definition of severe infection with H1N1pdm in China is similar to that in World Health Organization (WHO) Guidelines for Pandemic Influenza A(H1N1) 2009 and other Influenza Viruses (http://www.who.int/csr/resources/publications/swineflu/h1n1_guidelines_pharmaceutical_mngt.pdf?ua=1).

### Statistical analysis

Descriptive statistics comprised the calculation of median and interquartile ranges (IQRs) for continuous variables and absolute numbers and proportions for categorical variables. The demographic characteristics (age, gender and BMI), chronic medical conditions (including pulmonary, cardiovascular, metabolic and hematologic disorder; chronic kidney and liver disease; immune suppression; neural and muscular dysfunction), treatment (neuraminidase inhibitor, antibiotics and glucocorticoid administration) and clinical outcome were compared between severe patients with H7N9 and H1N1pdm. Several selected time durations associated with disease severity [7], i.e. interval from illness onset to medical consultation, neuraminidase inhibitor administration, hospitalization and death, were computed according to the recorded dates from the epidemiological investigations and medical records, and compared between severe patients infected with H7N9 and H1N1pdm virus. Pearson chi-square test was used for comparing proportions and continuity correction or Fisher’s Exact Test was used if appropriate. Mann-Whitney U test was used for comparing medians due to the small size of severe H7N9 patients. In adjusting comparisons, age and gender were adjusted using General Linear Model (for BMI) or Cox proportional hazards model (for selected time durations) for continuous variables and Logistic regression model for categorical variables. Statistical significance level was set at ≤0.05. Statistical analyses were conducted by R version 3.0.2. Statistical powers were computed by Power Analysis & Sample Size software (PASS, http://www.ncss.com/software/pass/) using the observed values of each selected variable in this study (excluding variables regarding treatment and other disorders in chronic medical conditions, because their differences between groups were no more than 0.015) (Table A in S1 File).

### Missing data

Patients’ clinical outcome was defined as “unrecovered” when they were discharged against medical advice, and they were considered as missing data in the context of the variable “death”. There were no missing data in age, gender and BMI. Information of missing data was shown in detail in Table B in S1 File. All the cases with missing data were excluded when selected variables being analyzed.

## Results

**Fig 1:**
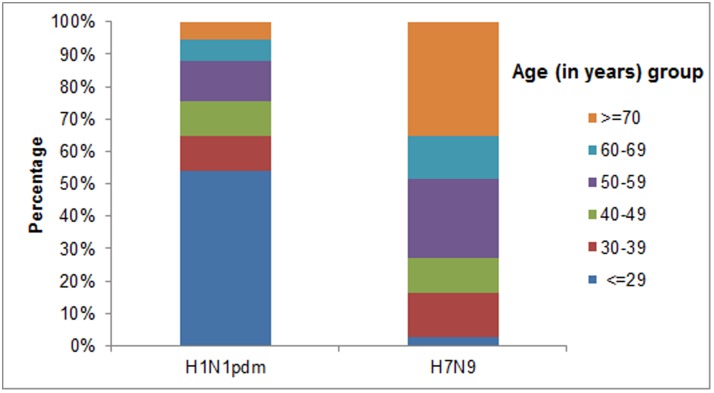
Age distribution of severe patients infected with influenza H7N9 and H1N1pdm in Jiangsu Province, China.

The median age of severe H7N9 patients was significantly older than that of severe H1N1pdm patients (59.0 vs.27.0 years, *P* < 0.0001). Patients older than 70 years accounted for a proportion of 35.1% for H7N9 but only of 5.4% for H1N1pdm (Fig. 1). Male accounted for a proportion of 73.0% in severe H7N9 patients whereas only 48.4% in severe H1N1pdm patients. No significant difference in BMI was observed between those two groups of patients (median, 23.9 vs. 22.9, ***P*** = 0.084). Severe H7N9 patients were more likely to have at least one chronic medical disorder (55.6%) than severe H1N1pdm patients (34.5%) (***P*** = 0.018). Cardiovascular disorders (excluding hypertension) were most frequently reported (25.0%) in severe H7N9 patients but not in severe H1N1pdm patients (7.3%) (***P*** = 0.004). Pulmonary disorders (including asthma), metabolic disorders (including diabetes mellitus) and other chronic disorders were also reported more frequently in severe H7N9 patients than in severe H1N1pdm patients, however, without any statistically significant differences. The pharmaceutical interventions in both types of cases were observed to be similar. Most of the patients of both H7N9 and H1N1pdm were treated with neuraminidase inhibitors, antibiotics and glucocorticoid, however, the clinical outcome was quite different. Nearly 85% of severe H7N9 patients were admitted to intensive care unit (ICU) compared with a proportion of 65.2% in severe H1N1pdm patients (***P*** = 0.026). Complications including acute respiratory distress syndrome (ARDS), respiratory failure, liver and renal dysfunction occurred more frequently in severe H7N9 patients, resulting in a significantly higher fatality rate than in severe H1N1pdm patients (45.7% vs. 15.3%, ***P*** < 0.0001). The most common complications were ARDS and respiratory failure in both H7N9 and H1N1pdm patients. The time intervals (days) from the onset of illness to first medical consultation, to hospitalization, to neuraminidase inhibitors administration and to death were significantly longer in severe H7N9 patients (Table 1).

**Table 1:** Distribution of selected variables between severe H7N9 and H1N1pdm patients.

Selected variables	H7N9 ^a^	H1N1pdm ^a^	***P*** ^b^	Adjusted ***P*** ^c^
Median age by years (IQR ^d^)	59.0 (46.5–72.5)	27.0 (7.00–48.8)	**<0.0001**	NA
Male, FREQ ^d^ (%)	27 (73.0)	89 (48.4)	**0.006**	NA
Median BMI ^d^ (IQR)	23.9 (22.7–25.3)	22.9 (19.4–25.5)	0.084	0.499
**Chronic medical condition,** FREQ (%)				
Pulmonary disorders (including asthma)	6 (16.7)	17 (9.60)	0.336	0.127
Cardiovascular disorders (excluding hypertension)	9 (25.0)	13 (7.30)	**0.004**	0.801
Metabolic disorders (including diabetes mellitus)	5 (13.9)	11 (6.20)	0.213	0.838
Other disorders	7 (19.4)	31 (17.9)	0.829	0.678
Any disorder	20 (55.6)	59 (34.5)	**0.018**	0.466
**Treatment,** FREQ (%)				
Neuraminidase inhibitor	27 (84.4)	153 (83.6)	0.913	0.571
Antibiotics	31 (96.9)	179 (97.8)	0.557	0.673
Glucocorticoid	23 (71.9)	132 (72.5)	0.939	0.592
**Clinical outcome,** FREQ (%)				
ICU admission	28 (84.8)	120 (65.2)	**0.026**	0.245
ARDS	23 (71.9)	80 (44.4)	**0.004**	**0.004**
Respiratory failure	28 (87.5)	110 (60.1)	**0.003**	0.064
Heart failure	9 (28.1)	31 (17.0)	0.138	0.132
Liver dysfunction	14 (43.8)	33 (18.5)	**0.002**	**0.009**
Renal dysfunction	14 (43.8)	18 (10.0)	**<0.0001**	**0.004**
Death	16 (45.7)	27 (15.3)	**<0.0001**	**0.011**
**Time duration**				
Interval from onset of illness to neuraminidase inhibitor treatment, Median days (IQR)	9.00 (5.00–13.0)	5.00 (3.00–8.00)	**<0.0001**	**0.014**
Interval from onset of illness to first medical consultation, Median days (IQR)	3.00 (1–6.00)	1.00 (0–4.00)	**0.002**	0.108
Interval from onset of illness to hospitalization, Median days (IQR)	6.00 (4.00–8.50)	4.00 (1.00–6.00)	**<0.0001**	0.082
Interval from onset of illness to death, Median days (IQR)	24.5 (20.0–37.3)	13.0 (7.00–22.0)	**0.001**	**0.019**

^a^ Patients with missing data were excluded for each variable.

^b^ Pearson chi-square test was used for comparing proportions and continuity correction or Fisher’s Exact Test was used if appropriate. Mann-Whitney U test was used for comparing medians.

^c^ Age and gender were adjusted using General Linear Model (for BMI) or Cox proportional hazards model (for selected time durations) for continuous variables and Logistic regression model for categorical variables.

^d^ Interquartile range (IQR), Frequency (FREQ), Body mass index (BMI)

Interestingly, after adjusting age and gender, no difference in chronic medical conditions remained significant between severe H7N9 and H1N1pdm patients. In the context of clinical outcome, ICU admission and respiratory failure became insignificant while ARDS, liver and renal dysfunction, and death remained significant between the severe H7N9 and H1N1pdm patients. In addition, only intervals from the onset of illness to neuraminidase inhibitor treatment and to death were still significantly longer in severe H7N9 patients than severe pH1N1 patients after adjusting age and gender (Table 1).

## Discussion

Most of the H7N9 patients were older male while most of the H1N1pdm patients were young without significant difference in gender [3, 8]. Significant differences were observed in age and gender between severe H7N9 and H1N1pdm patients in this study, which were consistent with another study conducted in patients hospitalized with H7N9 and H1N1pdm in China [9]. Chronic medical conditions are considered to be associated with the risk of hospitalization and prognosis of the patients infected with influenza virus [9], which need to be paid close attention by health care providers in prevention and treatment. Severe H7N9 patients were found more likely to have chronic medical conditions than severe H1N1pdm patients in gross comparison both in this study and a previously conducted study [9], however, the differences became insignificant after adjusting age and gender. This finding suggests that the differences in chronic medical conditions were probably due to the different demographic characteristics rather than susceptibility to severe infections with different influenza subtypes. No differences were found in treatment between H7N9 and H1N1pdm either before or after adjusting age and gender, which suggested that the combination of neuraminidase inhibitor, antibiotics and glucocorticoid was commonly applied for treating severe infections with both influenza subtypes, probably because of the high incidence of bacterial co-infection and its poor prognosis [10, 11].

Although the definitions of severe cases with H7N9 and H1N1pdm were comparable, a significantly worse clinical outcome was observed in H7N9 patients regardless of age and gender. Complications such as ARDS, liver and renal dysfunctions were much more frequent in severe H7N9 patients leading to a 3-fold case-fatality rate than severe H1N1pdm patients. These complications should be noticed to the clinicians owing to improve the clinical outcomes of H7N9 patients.

Time interval from onset of illness to first medical consultation was associated with severity of influenza infection [12]. Patients’ ages and influenza subtypes could influence the time intervals [13]. This might be the reason why the difference in time interval of medical consultation became insignificant after adjusting age and gender in our study. It seems that the two influenza virus subtypes has no obvious influence on infected patients’ medical consultation behaviors. The interval from onset of illness to neuraminidase inhibitor treatment was found significantly longer in severe H7N9 patients regardless of age and gender. Neuraminidase inhibitors treatment were commonly applied clinically during the H1N1pdm-driven pandemic period but has not been recommended to be widely used for outpatients with influenza like illness (ILI) in China. Thus, most H7N9 patients received neuraminidase inhibitors treatment when confirmed to be H7N9 cases or considered to be probable H7N9 cases, which led to a significantly longer interval from illness onset to anti-influenza medication.

Wang C et al found that the median time from onset to death was 18 days and 15 days for hospitalized H7N9 and H1N1pdm patients, respectively [9]. In our study, the time intervals were 24.5 days and 13.0 days for severe H7N9 patients and H1N1pdm patients. The differences between studies might be due to the various medical levels related to the geographic locations of the study populations. The interval from onset of illness to death was significantly longer in severe H7N9 patients even after adjusting age and gender, which suggested that the disease course of severe infections with H7N9 and H1N1pdm was different.

Although all the eligible cases in Jiangsu Province of China during the study period were included in this study, the sample size was small, which limited the statistical power. However, an average power of 0.765 (range, 0.279–1.000) was achieved, which was computed by Power Analysis & Sample Size software (PASS, http://www.ncss.com/software/pass/) using the observed values of each selected variable in this study (excluding variables regarding treatment and other disorders in chronic medical conditions, because their differences between groups were no more than 0.015). In fact, the statistical power was more than 0.80 and 0.60 in comparisons of 55.6% and 83.3% of selected variables, respectively (Table A in S1 File).

It has been reported that age and gender are associated with patients’ chronic medical conditions and clinical outcomes [14, 15]. Our results suggests that age and gender should be adjusted as important confounding factors when comparing the clinical features between severe H7N9 and H1N1pdm patients to avoid any misunderstanding regarding the differences between these two diseases particularly in terms of clinical severity and prognosis.

## Supporting Information

S1 File Table A. Statistical powers in comparisons of selected variables. Table B. Information of missing data. (DOCX)
