# Supplementary material for: Age and Gender Adjusted Comparison of Clinical Features between Severe Cases Infected with H7N9 and H1N1pdm Influenza A in Jiangsu Province, China
Source: PLoS One. 2015 Mar 27;10(3):e0120999. doi: 10.1371/journal.pone.0120999 (PMC4376887; doi:10.1371/journal.pone.0120999)
Supplement: S1 File — (DOCX) [file pone.0120999.s001.docx]

**Table A**. Statistical powers in comparisons of selected variables.

| Selected variables | H7N9 | H1N1pdm | Power ^a^ |
| --- | --- | --- | --- |
| Median age by years (IQR ^b^) | 59.0 (46.5-72.5) | 27.0 (7.00-48.8) | 1.000 |
| Male, Frequency (%) | 27 (73.0) | 89 (48.4) | 0.803 |
| Median BMI ^c^ (IQR) | 23.9 (22.7-25.3) | 22.9 (19.4-25.5) | 0.632 |
| **Chronic medical condition,** FREQ (%) |  |  |  |
| Pulmonary disorders (including asthma) | 6 (16.7) | 17 (9.60) | 0.279 |
| Cardiovascular disorders (excluding hypertension) | 9 (25.0) | 13 (7.30) | 0.828 |
| Metabolic disorders | 5 (13.9) | 11 (6.20) | 0.397 |
| (including diabetes mellitus) |  |  |  |
| Other disorders | 7 (19.4) | 31 (17.9) | -- ^d^ |
| Any disorder | 20 (55.6) | 59 (34.5) | 0.672 |
| **Treatment,** FREQ (%) |  |  |  |
| Neuraminidase inhibitor | 27 (84.4) | 153 (83.6) | -- |
| Antibiotics | 31 (96.9) | 179 (97.8) | -- |
| Glucocorticoid | 23 (71.9) | 132 (72.5) | -- |
| **Clinical outcome,** FREQ (%) |  |  |  |
| ICU admission | 28 (84.8) | 120 (65.2) | 0.679 |
| ARDS | 23 (71.9) | 80 (44.4) | 0.884 |
| Respiratory failure | 28 (87.5) | 110 (60.1) | 0.947 |
| Heart failure | 9 (28.1) | 31 (17.0) | 0.366 |
| Liver dysfunction | 14 (43.8) | 33 (18.5) | 0.888 |
| Renal dysfunction | 14 (43.8) | 18 (10.0) | 0.993 |
| Death | 16 (45.7) | 27 (15.3) | 0.970 |
| **Time duration** |  |  |  |
| Interval from onset of illness to neuraminidase inhibitor treatment, Median days (IQR) | 9.00 (5.00-13.0) | 5.00 (3.00-8.00) | 0.969 |
| Interval from onset of illness to first medical consultation, Median days (IQR) | 3.00 (1-6.00) | 1.00 (0-4.00) | 0.740 |
| Interval from onset of illness to hospitalization, Median days (IQR) | 6.00 (4.00-8.50) | 4.00 (1.00-6.00) | 0.724 |
| Interval from onset of illness to death, Median days (IQR) | 24.5 (20.0-37.3) | 13.0 (7.00-22.0) | 0.999 |

^a^ Computed by Power Analysis & Sample Size software (PASS) using the observed values of each selected variable in this study

**^b^** interquartile range (IQR)

^c^ body mass index (BMI)

d Statistical powers were not computed for these variables because their differences between groups were no more than 0.015.

**Table B**. Information of missing data

| **Selected variables** | **Number of missing data** | |
| --- | --- | --- |
|  | H7N9 (n=37) | H1N1pdm (n=184) |
| **Chronic medical condition** |  |  |
| Pulmonary disorders (including asthma) | 1 | 6 |
| Cardiovascular disorders (excluding hypertension) | 1 | 7 |
| Metabolic disorders(including diabetes mellitus) | 1 | 7 |
| Other disorders | 1 | 11 |
| At least one disorder | 1 | 13 |
| **Treatment** |  |  |
| Neuraminidase inhibitor | 5 | 1 |
| Antibiotics | 5 | 1 |
| Glucocorticoid | 5 | 2 |
| **Clinical outcome** |  |  |
| ICU admission | 4 | 0 |
| ARDS | 5 | 4 |
| Respiratory failure | 5 | 1 |
| Heart failure | 5 | 2 |
| Liver dysfunction | 5 | 6 |
| Renal dysfunction | 5 | 4 |
| Death | 1 | 8 |
| **Time duration** |  |  |
| Interval from illness onset to neuraminidase inhibitor treatment | 0 | 3 |
| Interval from illness onset to first medical consultation | 1 | 22 |
| Interval from illness onset to hospitalization | 0 | 6 |
| Interval from illness onset to death | 0 | 0 |
